# Dread of uncertain pain: An event-related potential study

**DOI:** 10.1371/journal.pone.0182489

**Published:** 2017-08-23

**Authors:** Yujing Huang, Qian Shang, Shenyi Dai, Qingguo Ma

**Affiliations:** 1 School of Economics and Management, Zhejiang Sci-tech University, Hangzhou, Zhejiang Province, People's Republic of China; 2 School of Management, Hangzhou Dianzi University, Hangzhou, Zhejiang Province, People's Republic of China; 3 School of Management, China Jiliang University, Hangzhou, Zhejiang Province, People's Republic of China; 4 School of Management, Zhejiang University, Hangzhou, Zhejiang Province, People's Republic of China; 5 Neuromanagement Laboratory, Zhejiang University, Hangzhou, Zhejiang Province, People's Republic of China; Erasmus University Rotterdam, NETHERLANDS

## Abstract

Humans experience more stress about uncertain situations than certain situations. However, the neural mechanism underlying the uncertainty of a negative stimulus has not been determined. In the present study, event-related potential was recorded to examine neural responses during the dread of unpredictable pain. We used a cueing paradigm in which predictable cues were always followed by electric shocks, unpredictable cues by electric shocks at a 50/50 ratio and safe cues by no electric shock. Visual analogue scales following electric shocks were presented to quantify subjective anxiety levels. The behavioral results showed that unpredictable cues evoked high-level anxiety compared with predictable cues in both painful and unpainful stimulation conditions. More importantly, the ERPs results revealed that unpredictable cues elicited a larger P200 at parietal sites than predictable cues. In addition, unpredictable cues evoked larger P200 compared with safe cues at frontal electrodes and compared with predictable cues at parietal electrodes. In addition, larger P3b and LPP were observed during perception of safe cues compared with predictable cues at frontal and central electrodes. The similar P3b effect was also revealed in the left sites. The present study underlined that the uncertain dread of pain was associated with threat appraisal process in pain system. These findings on early event-related potentials were significant for a neural marker and development of therapeutic interventions.

## Introduction

Dread of negative stimulation plays a central role in allowing an individual to prepare for and prevent aversive outcomes. The acquisition of this ability can be incorporated into a conditioning process [1–5] in which participants learn the association between an unconditioned stimulus (a negative stimulation) and a conditioned stimulus (a cue). The majority of previous evidence [6–8] indicated that perception of unpredictable cues evoked stronger anxiety-like behaviors than perception of predictable cues. However, few studies [8–13] measured the specific neural correlates of uncertainty. For example, functional magnetic resonance imaging (fMRI) studies showed that dread of unpredictable pain activated brain areas involved in the affective/cognitive aspects of the pain experience and the selection of proper viscero-sensorimotor responses [9,10]. Brown et al. (2008) revealed that dread regarding expected heat intensity involved a cortical network commonly associated with attention [11].

In regard to neurophysiological studies, the event-related potential (ERP) technique has been widely employed to reveal coherent stimulus-related postsynaptic activity in the cortex with high temporal resolution [14]. Hence, ERP was a useful tool in the on-line examination of the time course of cortical activation for the dread of unpredictable pain. It was observed that a stimulus-preceding negativity (SPN) between 1000–2000 ms was more negative after uncertainty compared to certainty prior to experiencing a painful stimulation [15]. However, the researchers did not investigate whether dread of uncertain pain would be reflected by earlier brain potentials. Neurophysiological correlates of pain anticipatory processes, particularly during very early stages, are very important in the development of therapeutic interventions [16]. Earlier potentials such as P200, P3b or LPP are critical to observing healthy subjects' attention and alertness to noxious stimuli at the very beginning of dread. These early ERPs could potentially serve as biomarkers and may be related to cognitive-affective pathways. In contrast, SPN is usually detected before a painful stimulation, which may be associated with the affective-motivation anticipation [17]. The amplitudes of SPN may be related to primary sensory cortices and reflect a conservative response tendency. The visual ERP correlates of the emotionality of stimuli are reflected by a positive component between 200–400 ms (P200) [18]. Previous studies suggested that stimuli with negative emotionality elicited increased P200 amplitudes relative to stimuli with positive emotionality [19–20]. Additionally, a larger late positive potential (LPP) was observed during encoding of pictures signaling threats compared to safety [21]. Weymar et al. [22] and Ma et al. [23] obtained similar results by modulating the (LPP) in anticipation paradigms. Moreover, P3b was sensitive to the arousal value of stimulation, with smaller amplitudes for unpleasant pictures compared to pleasant pictures at fronto-central sites [24]. However, it is unknown whether the earlier potentials (P200, P3b, LPP) elicited by dread of unpredictable pain are different from that of predictable pain.

In the current study, we proposed that stronger negative emotions were spontaneously activated by uncertainty in the early stage of dread compared to certainty. We used a cueing paradigm in which predictable cues were always followed by electric shocks, unpredictable cues were followed by electric shocks at a 50/50 ratio and safe cues were not followed by an electric shock. Visual analogue scales were presented following the electric shocks to quantify subjective anxiety levels. We hypothesized that unpredictable cues would spontaneously arouse more negative emotions, resulting in higher amplitudes of P200 compared to predictable cues. In addition, we hypothesized that a smaller amplitudes of P3b and LPP were elicited by unpredictable or predictable cues compared with safe cues sites.

## Methods

### Subjects

Nineteen healthy right-handed subjects were recruited (19 males, mean age = 22.32 years, with an age range of 20–25 years). Handedness assessment was based on the questionnaire developed by Annett(1970) [25]. Two datasets were discarded because of excessive artifacts. All participants reported normal or corrected-to-normal vision and had no history of neurological or psychiatric disorders. All subjects provided informed consent, and the study was approved by the institutional ethical committee of the School of Management at Zhejiang University. All participants were paid for their participation. Research was carried out according to the principles of the Declaration of Helsinki.

### Equipment

All visual stimuli were presented on a 19-inch CRT computer monitor (1280*1024 pixels, 60HZ) connected to a 2 GHz Pentium computer. Stimulus presentation and data collection were controlled by Eprime software. The grey background of monitor was grayscale (R = 128, G = 128, B = 128). The black foreground was used (R = 0, G = 0, B = 0). The visual stimuli were digitally processed by Adobe Photoshop digital image manipulation software so that each stimulus had the same luminance (14cd/m2) and root mean square (RMS) contrast. Electric shocks were supplied by Electron-ESA stimulators. Stimulation electrodes were placed on the backs of the participants’ left hands. We used an electrical pain stimulus delivered by a novel bipolar concentric surface electrode (stimulation area: 20 mm^2^), which predominantly depolarizes Aδ-fibers placed on the dorsum of the left hand of the subject. The skin was sanitized before the electrode was attached with tape.

### Experimental procedures

Prior to formal experiment, shock intensity was calibrated to find out participants' maximum shock intensity. For the calibration process, the threshold was determined by giving pulse starting from 0 mA. Variation of the voltage was increased by 0.01mA each time. The duration of a pulse was 1 ms. The shocks were manually controlled by the experimenter. The participants notified the experimenter that they couldn't bear it anymore and the current amplitude was defined as their maximum stimulation intensity.

Each participant was instructed, "when you feel that you absolutely cannot bear any stronger shock, let us know- this will be set as your maximum. You will receive a shock of 75% of the maximum tolerable voltage in the experiment". The maximum tolerable electric strength ranged from 9 mA to 21 mA. There was no tissue damage during the test sessions.

Then, participants sat with the ERP recording and painful stimulation equipments. A cueing paradigm was used in which an electric shock might or might not be administered upon the presentation of a warning cue. Each trial began with a fixation cross (400–600 ms) in the center of the screen followed by a blank screen with a duration of 800–1000 ms. Subsequently, a cue was presented for 1000 ms. For unpredictable trials, the cue was an "?" (Unpredictable Cue, UC), which was followed by an electric shock at a 50/50 ratio. For predictable trials, the cue was a "√" (Predictable Cue, PC), which was always followed by an electric shock. For neutral trials, the cue was an "X" (Safe Cue, SC), which was never followed by an electric shock. The sequences of conditions were counterbalanced across participants in formal experiments. The cue picture was seen from a distance of 100 cm in the center and occupied a visual angle of 6.27°*6.27°. Then, a shock may be administered for 1 ms according to the different conditions. The voltage levels of the shocks were 75% of an individual's maximum tolerable voltage. After shocks, the cue stayed at the center of the screen for another 1000 ms. Then, a blank screen followed for a duration of 800–1000 ms. Visual analogue scales (VAS) (0 to 100; ends defined as 0: none to 100: very much) were presented for each trial to quantify subjective anxiety levels. Specifically, subjects were instructed, "Tell us how anxious this waiting was." The VAS disappeared after participants' responses.

Subjects were informed about all cue-shock pairings prior to the experiment. There were 50 trials for each type of cue. The trial order was pseudorandomized. The experiment consisted of 3 blocks, with 50 trials for each block. The duration of the experiment was approximately 15 min. Prior to the experimental blocks, participants performed one training block in order to familiarize themselves with the task.

### Electroencephalogram (EEG) recording

The EEG was recorded from a set of 64 Ag/AgCl electrodes according to the 10/20 system. The EEG was recorded in DC mode. The time constant of amplifier was 10s. ERP was filtered online with a bandpass of 0.05–100 Hz. The sampling rate was 500 Hz using the Neuroscan Synamp2 Amplifier (Neuroscan Labs, Sterling, VA, USA). The EEG eletrodes were on-line referenced to the left mastoid; and the right mastoid was actively recorded. Data were re-referenced offline to the average of both mastoids. The ground electrode was placed on the medial frontal location. The vertical electrooculogram (EOG) was recorded with electrodes placed on the supra-orbital and infra-orbital locations of the left eye. The horizontal EOG was recorded from electrodes on the outer canthi of both eyes. Electrode resistance was kept below 5kΏ.

### ERP data analysis

The ERP data, time-locked to the onset of cue pictures prior to painful stimulation, were analyzed offline. Neuroscan 4.3.1 software was used for EEG pre-processing. The ERP data were epoched from -200 to 1000 ms. Before averaging, trials with eye movement artifacts as well as peak-to-peak deflections exceeding ±80 μv were excluded. After exclusion of the trials with artifacts, the mean number of available trials per participant was 44 in UC conditions, 41 in PC conditions and 39 in SC conditions. The ERP was filtered offline with a low-pass filter of 30Hz (24dB/octave) with zero phase shift. Brain potentials were aligned to a 200 ms baseline.

We selected the following 9 electrodes for statistical analysis: F3, C3, P3 (left sites); Fz, Cz, Pz (midline sites); F4, C4, P4 (right sites). The latency window for P200 in the present study was similar to those used by Gole et al. [7], yielding 200–260 ms. The time window of P3b employed in the study was 400–700 ms. The time window of LPP was 700–1000 ms. Within each time window, the averaged potentials were fed into within-participant repeated measures of analysis of variance (ANOVA). ANOVA factors were *Cue* (UC, PC and SC), *Caudality* (frontal, central and parietal sites) and *Laterality* (left, midline and right sites). In the primary post-hoc analysis, we used post-hoc tests of repeated-measure to compare the differences in the LPP, P200 and P3b per the 3 *Caudality* levels. We also performed other post-hoc analyses to examine the mean difference per *Cue* and *Laterality* levels. The mean difference was significant at the 0.05 level. The statistical software was SPSS. The degree of freedom of the F-ratio was corrected according to the Greenhouse-Geisser method. Effect sizes were given as Cohen’s d values.

## Results

### Behavioral results

**Fig 1:**
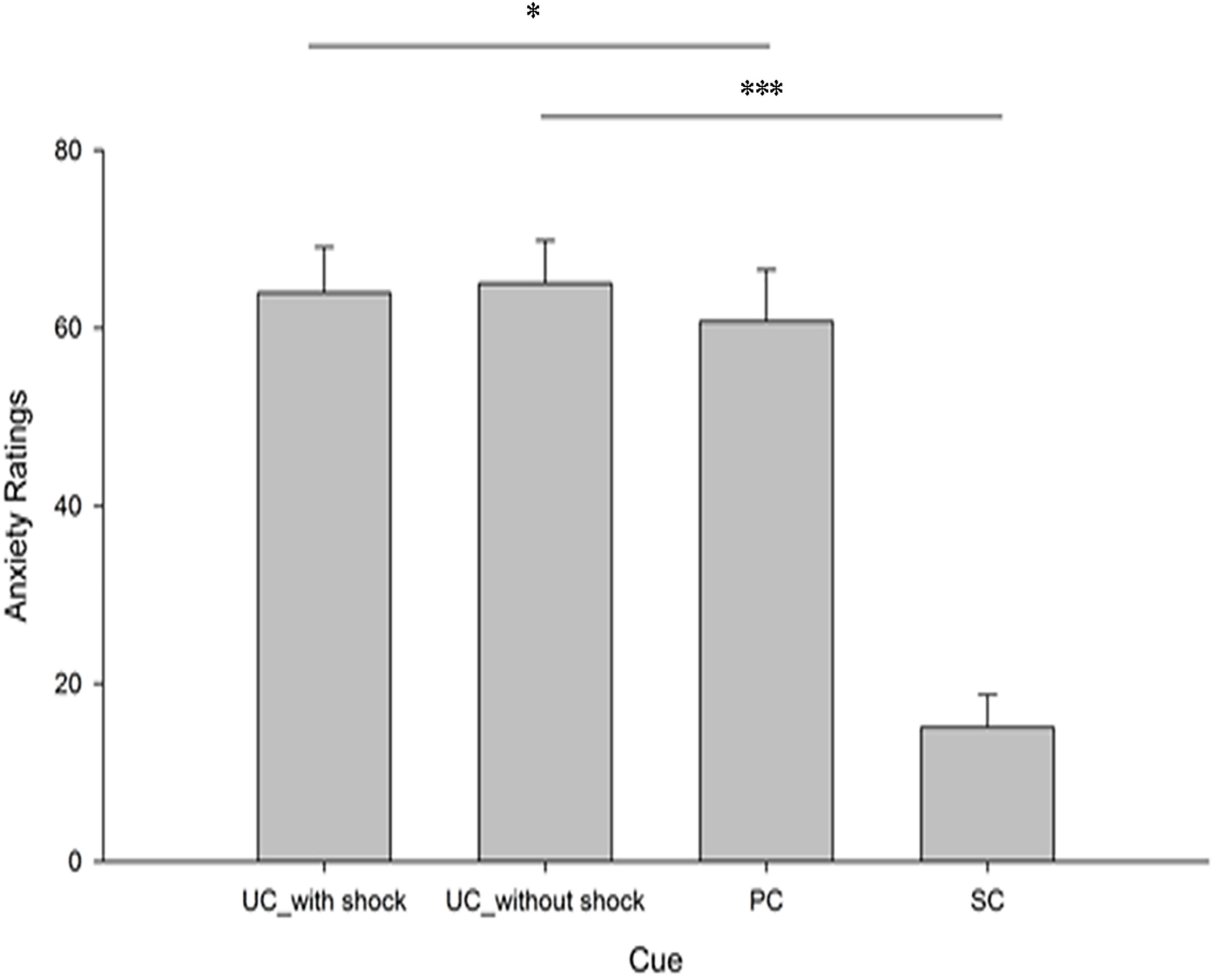
Mean subjective anxiety level ratings for cues (UC-with shock, UC-without shock, PC, SC). The x-axis indicates independent variables: UC-with shock, UC-without shock, PC versus SC. The y-axis indicates the mean anxiety level ratings by all subjects (mean±S.E.). Error bars depict the standard errors.

To control the influence of perceived pain on anxiety ratings, we divided UC trials into UC-with shock and UC-without shock trials. We used 2 (*Cue*: predictable or unpredictable) x 2 (*Stimulation*: painful or unpainful) repeated-measures ANOVA to analyze the ratings. The analysis revealed a significant main effect of *Cue*, F(1,16) = 85.69, p < 0.001 (two-tailed), effect size = 2.31, and a main effect of *Stimulation*, F(1,16) = 39.23, p < 0.001(two-tailed), effect size = 1.56. There was a significant interaction effect between *Cue* and *Stimulation*, F(1,16) = 45.74, p < 0.001(two-tailed), effect size = 1.69. Post-hoc analyses found that anxiety ratings following the painful stimulation with participants in the unpredictable cue conditions (mean = 65.01, S.E. = 5.11) were significantly higher than participants in predictable cue conditions (mean = 60.79, S.E. = 5.79) (p = 0.04). A similar result was shown that anxiety ratings following the unpainful stimulation with participants in the unpredictable cue conditions (mean = 63.98, S.E. = 4.81) were significantly higher than participants in predictable cue conditions (mean = 15.12, S.E. = 3.11) (p < 0.001). Thus, cue images were effective in moderating anxiety levels. The results are shown in Fig 1.

### ERP results

**Fig 2:**
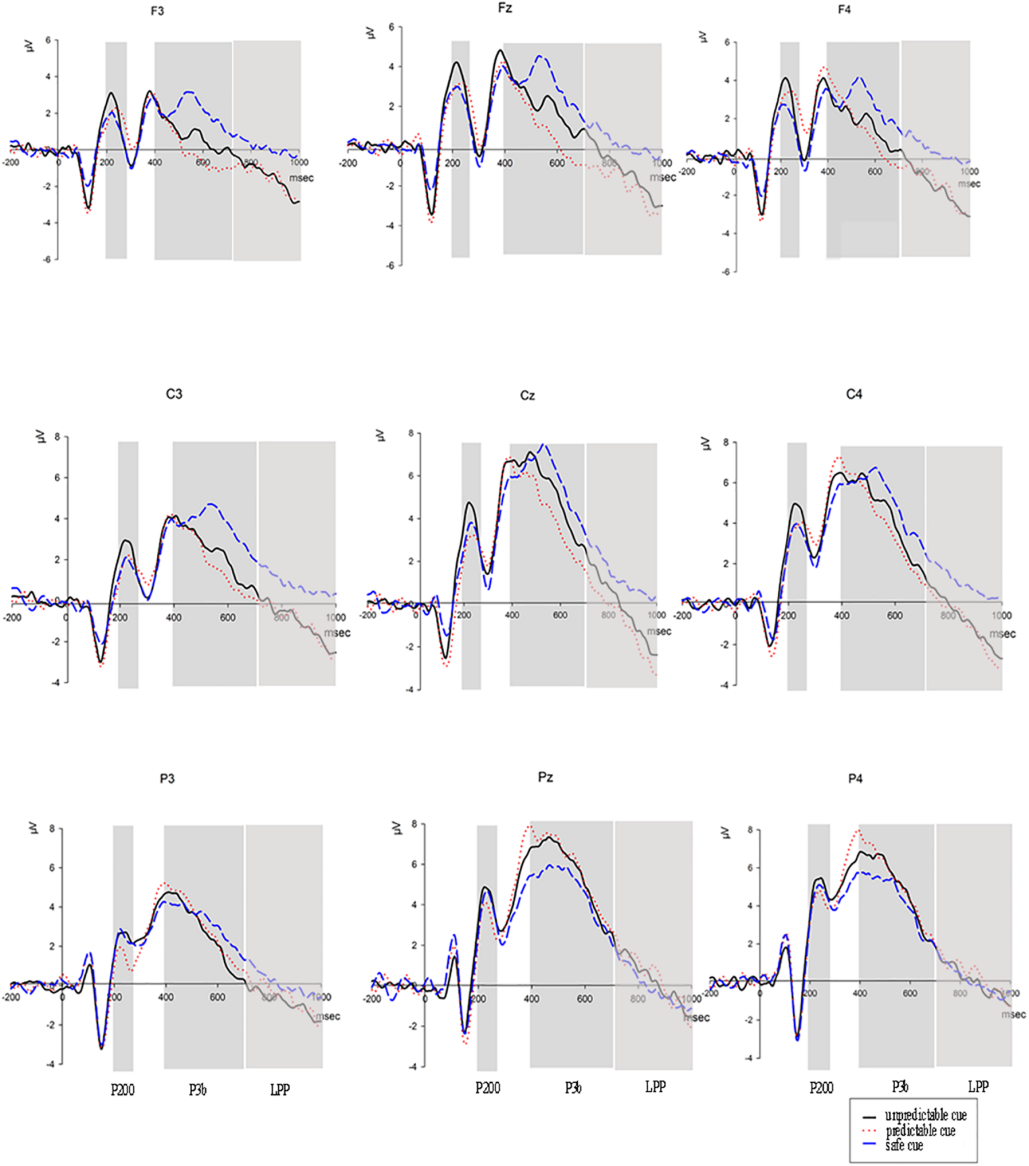
Grand-averaged ERP waveforms at nine electrodes evoked by cue pictures. The black line indicates ERP waveforms evoked by UC, the long-dashed line illustrates ERP waveforms elicited by SC, the short-dashed line shows ERP waveforms in the PC condition. The x-axis indicates the time scale, which is marked in intervals of 200 ms. The time scale started 200 ms pre-stimulus onset. The y-axis indicates the amplitudes, which are marked in intervals of 2μv.

As shown in Fig 2, the ANOVA results revealed that for the P200 time window, there was no significant main effect of *Cue* (F(2,32) = 2.35,p = 0.11,two-tailed,effect size = 0.38). However, a significant interaction effect between *Cue* and *Caudality* (F(4,64) = 3.20, p = 0.04, two-tailed, effect size = 0.45) was observed. Post-hoc comparisons revealed that P200 amplitudes were larger in unpredictable cue condition (mean = 3.20, S.E. = 1.00) compared with those in safe cue conditions (mean = 2.22,S.E. = 0.85) (p = 0.05) at frontal electrodes. Also, P200 amplitudes were larger in unpredictable cue conditions (mean = 4.01, S.E. = 0.62) compared to those in predictable cue conditions (mean = 3.13,S.E. = 0.73) (p = 0.029) at parietal electrodes. We found no significant interaction effect between *Cue* and *Laterality* (F(4,64) = 1.28, p = 0.29, two-tailed, effect size = 0.28), nor significant interaction effect among *Cue*, *Caudality* and *Laterality* (F(8,128) = 1.46, p = 0.18, two-tailed, effect size = 0.30). The amplitudes of P200 at nine sites are shown in Table 1.

**Table 1:** Amplitudes of P200 at nine electrodes.

	Mean±S.E.		
	UC	PC	SC
F3	2.50±0.92	2.04±0.82	1.66±0.84
Fz	3.53±1.15	3.05±1.02	2.59±0.93
F4	3.55±0.98	3.29±0.89	2.40±0.83
C3	2.55±0.63	2.01±0.64	1.74±0.54
Cz	4.26±0.95	3.00±0.93	3.43±0.81
C4	4.59±0.72	3.76±0.74	3.65±0.68
P3	2.50±0.61	1.53±0.75	2.43±0.64
Pz	4.43±0.77	3.50±0.82	4.08±0.67
P4	5.07±0.81	4.35±0.66	4.66±0.84

Potentials (expressed in μv) recorded at nine electrodes for unpredictable cues (UC), predictable cues (PC) and safe cues (SC) in the time window of 200–260 ms. The standard errors are reported.

As for P3b, no main effect of *Cue* (F(2,32) = 1.53, p = 0.23, two-tailed, effect size = 0.31) was found. However, a significant interaction effect between *Cue* and *Caudality* (F (4,64) = 9.29, corrected p<0.001, two tailed, effect size = 0.76) was observed. In addition, we found a significant interaction effect between *Cue* and *Laterality* (F(4,64) = 3.25, p = 0.017, two-tailed, effect size = 0.45) and a significant interaction effect among *Cue*, *Caudality* and *Laterality* (F(8,128) = 2.39, p = 0.019, two- tailed, effect size = 0.38). Post-hoc analyses demonstrated that safe cues (mean = 2.75,S.E. = 1.20) elicited a more positive P3b than predictable cues (mean = 0.90,S.E. = 1.28) (p = 0.006) at frontal sites. In addition, safe cues (mean = 4.91,S.E. = 0.99) elicited larger P3b than predictable cues (mean = 3.35, S.E. = 1.03) at central electrodes (p = 0.01). As regard to the interaction effect between cue and laterality, we observed that larger amplitudes of P3b elicited by safe cues (mean = 2.97, S.E. = 0.88) were significant than those elicited by predictable cues (mean = 1.58, S.E. = 0.89) at left sites (p = 0.008). The P3b differences between predictable cues and safe cues were not significant at right sites and middle sites (all p > 0.05). There were no significant differences of P3b between predictable and unpredictable cues at any laterality (all p > 0.05). The amplitudes of P3b between unpredictable cues and safe cues were also not significant at any laterality (all p > 0.05). The amplitudes of P3b at nine sites are shown in Table 2.

**Table 2:** The amplitudes of P3b at nine sites.

	Mean±S.E.		
	UC	PC	SC
F3	0.87±0.91	0.12±1.31	2.17±1.24
Fz	2.09±1.02	1.03±1.39	3.18±1.25
F4	1.76±0.95	1.54±1.23	2.88±1.17
C3	2.06±0.68	1.59±0.95	3.48±0.93
Cz	5.31±0.97	4.28±1.19	5.92±1.07
C4	4.57±0.89	4.17±1.17	5.31±1.09
P3	2.70±0.76	3.01±0.85	3.23±0.78
Pz	5.57±0.98	5.81±1.03	4.78±0.81
P4	4.94±0.71	5.11±0.77	4.31±0.72

Potentials (expressed in μv) recorded at the frontal, central and parietal electrodes for unpredictable cues (UC), predictable cues (PC) and safe cues (SC) in the time window of 400–700 ms. The standard errors are reported.

As regard to LPP, there was no main effect of *Cue* (F(2,32) = 3.05, p = 0.06, two-tailed, effect size = 0.44). However, an interaction effect between *Cue* and *Caudality* was recorded (F(4,64) = 3.62, p = 0.03, two-tailed, effect size = 0.48). Post-hoc analyses indicated that the amplitudes of LPP were largest in safe cue conditions (mean = 0.25,S.E. = 0.94) compare with those in predictable cue conditions (mean = -1.61,S.E. = 1.29) (p = 0.02) and those in unpredictable cue conditions (mean = -1.32, S.E. = 0.91) (p = 0.04) at frontal sites. In addition, the amplitudes of LPP were the most positive in safe cue conditions (mean = 1.10,S.E. = 0.85) compared to those in predictable cue conditions (mean = -0.87,S.E. = 0.93) (p = 0.002) and those in unpredictable cue conditions (mean = -0.57,S.E. = 0.70) (p = 0.03) at central electrodes. There were no interaction effect between *Cue* and *Laterality* (F(4,64) = 0.97, p = 0.43, two-tailed, effect size = 0.25) nor interaction effect among *Cue*, *Caudality* and *Laterality* (F(8,128) = 1.83, p = 0.08, two-tailed, effect size = 0.34). The amplitudes of LPP at nine sites are shown in Table 3.

**Table 3:** Amplitudes of LPP at nine electrodes.

	Mean±S.E.		
	UC	PC	SC
F3	-1.30±0.84	-1.46±1.24	0.18±0.91
Fz	-1.18±1.12	-1.93±1.53	0.16±1.04
F4	-1.46±0.93	-1.41±1.19	0.38±0.98
C3	-1.05±0.59	-1.00±0.84	0.78±0.73
Cz	-0.03±0.92	-0.75±1.14	1.35±0.92
C4	-0.63±0.85	-0.87±1.08	1.15±1.03
P3	-0.80±0.58	-0.55±0.68	0.14±0.66
Pz	0.35±0.86	0.45±0.91	0.02±0.57
P4	0.11±0.91	0.37±0.99	0.09±0.89

Potentials (expressed in μv) recorded at the frontal, central and parietal electrodes for unpredictable cues (UC), predictable cues (PC) and safe cues (SC) in the time window of 700–1000 ms. The standard error is reported.

## Discussion

Our study illustrated four key findings. First, a robust behavioral effect was shown; subjective anxiety levels were higher when cues signaling a threat (i.e., unpredictable cues) were presented compared to the presentation of predictable cues when anxiety ratings were followed by the painful and unpainful stimulation. Second, unpredictable cues evoked larger P200 amplitudes at parietal sites compared to predictable cues and larger P200 amplitudes than safe cues at frontal sites. Third, the amplitudes of P3b for safe cues were larger than predictable cues at frontal and central sites. Fourth, safe cues evoked the largest amplitudes of LPP compared to predictable cues and unpredictable cues at the frontal and central sites.

According to behavioral results, uncertain dread can lead to stronger anxiety than predictable dread. In line with our findings, a previous study on patients with Crohn's disease[26] suggested that anticipation and uncertainty can lead to anxiety. Additionally, Rubio et al. (2015) [27] demonstrated that the uncertainty of the occurrence, timing and intensity of an aversive event may increase anticipatory anxiety. We suggested that increased anxiety responses to unpredictable threats may be related to automatic threat appraisal processes, which are associated with a brain circuit involving sensory, cognitive and emotional aspects [28]. If given a signal of threat, individuals assess the likelihood and strength of aversiveness for a threat avoidance strategy. This explanation was supported by Healthcote and Eccleston's study[29]. They proposed a cognitive-affective model to explain the uncertain threat of disease recurrence.

Our ERP data showed that a larger P200 occurred during the early phase in the uncertain condition compared with the certain condition at the parietal areas. Although the meanings of P200 were functionally and topographically controversial [30], there may be four explanations for the amplitudes of P200. First, automatic attention capture for negative stimuli is a possibility for P200 at centro-parietal or occipital sites [31,32]. Second, the P200 at frontal/parietal sites may be viewed as classification processes [33]. Third, P200 effect may indicate predictability effects or frequency effects, that is, a larger P200 effect in the high-frequency range [34]. Fourth, modulation of P200 amplitudes may reflect the early evaluation of affective connotation [35]. According to our findings, the P200 effect at parietal sites may be cautiously interpreted as an early stage of emotional evaluation.

LPP or P3b amplitudes were more positive in response to safe cues compared with cues signaling a threat (i.e., unpredictable and predictable cues) at frontal and central sites. Later positive components shift typically start at approximately 300 ms and last until 1000 ms [36]. Its amplitudes are sensitive to various aspects of stimuli, including probability of occurrence, high-level motivational evaluation and attention allocation [37,38]. However, several studies [39–42] presented a modulation of late positive potentials by the emotionality of stimuli. First, Bass et al. (2002) [40] confirmed that threat relative to safe cues elicited fear and increased P300. The cortical responses may be related to attentional selection models and emotional processing. Second, Mehmood et al. (2016) [41] exhibited an increased LPP when viewing positive images. The increased LPP effect was interpreted as the detection of emotion regulation, especially in children. Third, Schupp et al. (2004) [42] demonstrated that affective pictures (i.e., pleasant and unpleasant pictures) elicited enlarged late positive potentials over centro-parietal sensor sites than neutral pictures. It reflected a quick glimpse of emotionality of stimuli to tune the brain for selective perceptual processing. Fourth, Martin et al. (2017) [43] exhibited an increased LPP when passively viewing positive images as well as an atypical decreased LPP when increasing positive emotion. Their results could be associated with the unique automatic and controlled emotional abnormalities in schizophrenia-spectrum personality disorders. In our study, the enlarged late positive potentials at frontal/central sites may represent an emotional appraisal processing.

This study had several aspects that need to be improved in future. The current study may not tease the effects of the complexity, luminance and familiarity of cue images in experimental design. Thus, pairings of cues and stimulation need to be counterbalanced in design in future studies. As regard to the stimulation voltage, participants received a shock of 75% of the maximum tolerable voltage but it is recommended to use individual difference values of minimum and maximum tolerable pain intensity [44] in design. Thus, it is difficult to judge whether the anxiety levels and ERP results are subject to differences in personality or leisure activities or not.

In conclusion, our data provide insight into how the brain encodes unpredictability during dread of painful stimuli. Our behavioral findings confirm the pivotal role of unpredictable signals in anxiety-like responses. We found that an anticipatory P200 at parietal sites allowed for discrimination between uncertain and certain dread of pain. In addition, we presented evidence that late positive potentials amplitudes at the frontal and central sites were higher in safe conditions compared to threat conditions. These findings emphasize the importance of uncertain and anticipation on the threat appraisal in pain system.

## Supporting information

S1 Table Behavioral and ERP datasets analyzed in the manuscript. This file listed behavioral data and mean amplitudes of P200, P3b, LPP.
(XLSX)

S2 Table Behavioral and ERP datasets discarded. This file listed two participants data which were discarded. The behavioral data and mean amplitudes of P200, P3b, LPP were presented.
(XLSX)
